# Analysis of biosynthetic genes of phenolamides in *Ricinus communis* L. based on metabolomics and transcriptomes

**DOI:** 10.3389/fpls.2026.1836215

**Published:** 2026-05-25

**Authors:** Hua Li, Xiaojia Zhang, Sishu Huang, Saiwen Li, Congping Xu, Chuansong Zhan, Yanping Luo

**Affiliations:** 1 School of Tropical Agriculture and Forestry, School of Ecology, Hainan University, Haikou, China; 2 Yazhouwan National Laboratory (YNL), Sanya, China; 3 School of Life Science and Technology, Wuhan Polytechnic University, Wuhan, China

**Keywords:** biosynthesis, hydroxycinnamoyl transferase, phenolamides, *RcHCT1* and *RcHCT2*, *Ricinus communis* L.

## Abstract

*Ricinus communis* L., a medicinal plant widely distributed in tropical regions, is recognized not only for its use in traditional medicine but also for its antioxidant and other bioactive properties, which are largely associated with phenolamides. But the biosynthetic pathways of phenolamides in castor remain inadequately characterized. An integrated metabolomic and transcriptomic study was conducted to investigate the biosynthesis of phenolamides in root, stem, and leaf tissues at three time points (06:00, 12:00, and 18:00). The findings revealed that N-feruloyl putrescine was the predominant phenolamide, exhibiting peak accumulation in root tissues at 06:00. Integrated omics analysis and subsequent *in vitro* enzymatic assays led to the identification and functional characterization of two novel BAHD hydroxycinnamoyl transferase, *RcHCT1* and *RcHCT2*. And *RcHCT1* displayed broad substrate specificity, catalyzing the formation of N-feruloyl putrescine, N-caffeoyl putrescine, p-coumaroyl putrescine, N-caffeoyl serotonin, and N-feruloyl agmatine from the corresponding substrates: putrescine, serotonin, and agmatine. In contrast, *RcHCT2* exhibited narrower catalytic activity, producing only N-feruloyl putrescine and N-caffeoyl serotonin from putrescine and serotonin. Expression of both genes was also highest in roots at 06:00. And subcellular localization of *RcHCT1* and *RcHCT2* indicates that both proteins were localized to both the nucleus and the cytoplasm. In addition, the active pockets of *RcHCT1* and *RcHCT2* binding to different substrates, as well as adjacent amino acid residues, were excavated through molecular docking models. In conclusion, these findings provide critical insights into the biosynthetic pathway of phenolamides in castor, opening new avenues for its utilization in the design of bioactive molecules.

## 1 Introduction

Phenolamides, also known as hydroxycinnamic acid amides HCAAs or benzoylamides, are a class of specialized metabolites widely distributed in the plant kingdom (Herrmann and Nagel, 1989). Structurally, they are characterized by the presence of at least one hydroxycinnamic acid derivative linked to aromatic monoamines or aliphatic polyamines through amide bonds. Research on the biological activities of phenolamides began in the 1970s, primarily in the context of ethnopharmacology (Funayama et al., 1980). In fact, various medicinal herbs and berries are rich in phenolamides. With advancements in separation and purification technologies, an increasing number of phenolamides compounds have been isolated and identified, often confirmed as active ingredients responsible for various health benefits. Among these, a review on the phytochemistry of Lycium plants reported 42 different phenolamides, accounting for approximately 12% of the known metabolites in the genus (Dan et al., 2017). Phenolamides exhibit a broad range of health-related biological properties, including anti-inflammatory, antioxidant, antimicrobial, and anticancer activities, as well as protective effects against metabolic syndrome, cardiovascular diseases, and neurodegenerative disorders (Roumani et al., 2020).

Research has demonstrated that phenolamides play significant roles in key biological processes, including plant development and defense mechanisms (Edreva et al., 2007). Certain phenolamides function as phytoalexins and contribute to plant resistance against pathogens (Park et al., 2009). Research by Kaur et al. further confirmed the function of phenolamides in insect resistance, demonstrating that transgenic plants lacking specific phenolamides (caffeoyl putrescine and dicaffeoyl spermidine) exhibited significantly reduced insect resistance (Kaur et al., 2010). Additionally, phenolamides are also believed to participate in plant defense responses to abiotic stresses, such as mineral deficiency, dehydration, salt stress, and ultraviolet radiation (Demkura et al., 2010; Königshofer and Lechner, 2002).

As a research focus in plant secondary metabolism, the biosynthesis of phenolamides is highly conserved across species, indicating significant evolutionary conservation (Bassard et al., 2010). The pathway is initiated by the key enzyme hydroxycinnamoyl transferase (HCT) (Negrel and Javelle, 1997). Various HCTs have been cloned and identified in different plants, including tyramine HCT in potato, tomato, and rice (Kang et al., 2009; Schmidt and Strack, 1998); putrescine HCT in rice and maize (Marti et al., 2013; Peng et al., 2016b; Tanabe et al., 2016); serotonin HCT in pepper (Sei Kang et al., 2006); tryptamine/tyramine HCT in rice (Dong et al., 2015; Peng et al., 2016b); and agmatine HCT in Arabidopsis (Atsushi et al., 2009). Additionally, branches I, IV, and V of the BAHD acyltransferase family contribute to phenolamides biosynthesis (Peng et al., 2016a). Specific examples include agmatine coumaroyl transferase in Arabidopsis and barley (Atsushi et al., 2009; Burhenne et al., 2003), spermine HCT in Arabidopsis, eggplant, and rice (Grienenberger et al., 2009; Luo et al., 2019; Peng et al., 2016a), and putrescine HCT reported in both dicotyledons (e.g., tobacco) and monocotyledons (e.g., rice) (Onkokesung et al., 2012).

The exploration of biosynthetic gene clusters (BGCs) using multi-omics techniques is a growing research focus (Zhan et al., 2022), with numerous reports on plant phenolamides BGCs (Cao et al., 2024; Shen et al., 2022, Shen et al., 2021). In tomato, two domestication-negative-selected BGCs (BGC7 and BGC11) and their positive regulator SlMYB13 were found to regulate phenolamides accumulation and drought tolerance (Cao et al., 2024). A unique hydroxycinnamoyl tyramine BGC identified in rice enhances disease resistance (Shen et al., 2021). In *Euphorbia lathyris*, a diterpenoid BGC containing ElBAHD16 and ElBAHD35 was identified (Zhao et al., 2025). Additionally, five acyltransferases in *E. peplus* were recently shown to modify ingenol scaffolds to produce ingenol 3-angle and ingenol 3-angle 20-acetate (Schotte et al., 2025).

As a member of the *Euphorbiaceae* family, *Ricinus communis* L. has been reported to possess phospholipid acyltransferases (Arroyo-Caro et al., 2013; Xu et al., 2020), yet no BAHD acyltransferases involved in polyamine modification have been identified. This study integrates transcriptomic and metabolomic analyzes to identify two hydroxycinnamoyl transferase of BAHD family responsible for phenolamide biosynthesis. These findings bridge a genetic gap in the understanding of phenolamides in castor and provide a theoretical foundation for enhancing the future utilization of phenolamide biosynthesis.

## 2 Materials and methods

### 2.1 Experimental materials

The castor plants (**Supplementary Figure 1**) and treatments required for this experiment are consistent with the references (Li et al., 2024). The castor plants utilized in this study were cultivated from wild seeds procured from Guizhou Xinyuan Seed Industry Company, China. After outdoor planting, the castor seedlings were allowed to grow until they developed 5–6 leaves (**Supplementary Figure 1**). At this stage, samples were harvested from the plants at three time points on the same day: 06:00, 12:00, and 18:00. Each sampling event involved six castor plants, from which the roots (R), stems (S), and leaves (L) were separately collected. Half of the plants were designated for metabolomic analysis, and the other half for transcriptomic analysis. All samples were immediately flash-frozen in liquid nitrogen and then stored at -80 °C. For each time point, three castor plants were sampled, and their roots, stems, and leaves were separated to create three biological replicates for UV-B-induced treatments. These samples were also flash-frozen in liquid nitrogen and stored at -80 °C.

### 2.2 Metabolomics analysis

Metabolomic analysis was performed using non-targeted instrumentation consisting of an Ultra Performance Liquid Chromatography system (UPLC, CBM 40A, Shimadzu, Japan) coupled with a Sciex ZenoTOF 7600 quadrupole-time-of-flight mass spectrometer (Sciex, Canada). Separation was carried out on a Waters Acquity UPLC HSS T3 C18 column (2.1 mm × 100 mm, 1.8 µm; Waters, USA) with a mobile phase composed of ddH_2_O with 0.04% acetic acid (A) and acetonitrile with 0.04% acetic acid (B). The gradient elution program was as follows: 5% B at 0 min, increased to 95% B over 10 min, held at 95% B from 10 to 12 min, returned to 5% B at 12.1 min, and maintained until 15 min. The flow rate was 0.35 mL/min, the column temperature was kept at 40 °C, and the injection volume was 5 µL.

Data acquisition was performed in positive ion mode using TOF MS-IDA. The ESI ion source parameters were set as follows: temperature, 550 °C; ion spray voltage I (GSI), 50 psi; ion spray voltage II (GSII), 60 psi; curtain gas (CUR), 35 psi; spray voltage (IS), 5500 V; declustering potential (DP), 80 V; collision energy (CE), 30 V; and collision energy spread (CES), 15 V. The mass scan ranges were 50-1000 Da for both primary and secondary mass spectrometry. Metabolite ion information was extracted from Q-TOF non-targeted data using AB Science MS Converter software for subsequent widely targeted metabolomic analysis.

Targeted detection was performed using an Ultra Performance Liquid Chromatography system (UPLC, CBM 40A, Shimadzu, Japan) coupled with a Sciex 6500+ QTRAP mass spectrometer (Sciex, Canada). The liquid phase conditions were consistent with those used in non-targeted detection: mobile phase A consisted of ddH_2_O with 0.04% acetic acid, and phase B was acetonitrile with 0.04% acetic acid. The flow rate was maintained at 0.35 mL/min. The gradient elution program was as follows: 5% B at 0 min, increasing to 95% B at 10 min, held at 95% B from 10 to 12 min, and returned to 5% B at 12.1 min until 15 min. The column temperature was set to 40 °C, and the injection volume was 2 μL.

Targeted detection was conducted using Multiple Reaction Monitoring Information Dependent Acquisition Enhanced Product Ions (MRM-IDA-EPI). The ESI ion source parameters were set as follows: temperature, 450 °C; GSI, GSII, and CUR at 50, 60, and 35 psi, respectively; positive ion mode ion spray voltage (IS): 5500 V; collision gas (CAD): high. Each MRM event had a dwell time of 5 milliseconds, with a total cycle time of 0.8 seconds. Raw data were processed using Analyst 1.7 software for metabolite confirmation, followed by chromatographic peak correction and integration in MultiQuant 3.0.3. Peak areas were quantified with the MQ4 algorithm, representing the relative metabolite content.

Metabolites were confirmed using authentic standards or putatively annotated based on spectral database matching. For standard-confirmed metabolites, authentic standards were analyzed under the same analytical conditions as the samples, and identification was based on the agreement of accurate m/z, retention time, and MS/MS fragmentation patterns. For metabolites without available standards, putative annotations were assigned by matching MS/MS spectra against an in-house library or public databases, including MoNA and METLIN, using a cosine similarity score > 0.7 and requiring at least three matched fragment ions.

### 2.3 Transcriptomics analysis

The transcriptome data of castor were consistent with a previous reference (Li et al., 2024). The reference genome of castor (*R. communis* v0.1) was sourced from Phytozome. Fragments per kilobase oftranscript permillionmapped reads (FPKM)were used to calculate the gene expression levels. Each organization had three replicates of FPKM data at each time point. StringTie software was used to quantify gene expression levels, and DESeq2 was employed to identify differentially expressed genes (DEGs). Orthogonal partial least squares-discriminant analysis (OPLS-DA) was applied to obtain VIP values for DEG screening. DEGs were identified across root, stem, and leaf tissues at 06:00, 12:00, and 18:00 using the criteria: padj ≤ 0.05 and |FoldChange| ≥ 2.

### 2.4 *In vitro* enzyme assays

Prokaryotic protein expression was performed as previously described (Li et al., 2024; Zhan et al., 2021). Briefly, 1 µL of the plasmid vector pGEX6p-1 carrying the target gene was added to 20 µL of *E. coli* BL21(DE3) expression competent cells. After incubation, the bacteria were collected and thoroughly resuspended. The bacterial suspension was lysed using a pre-chilled disruptor until clarity was achieved. The lysate was collected as crude protein. A portion of the crude protein was subjected to low-temperature centrifugation (4 °C, 13,000 rpm, 60 min) to obtain the soluble supernatant protein. All protein samples were stored at low temperature.

Protein purification was carried out according to previously described methods (Li et al., 2024; Zhan et al., 2023). Briefly, 40 mL of supernatant protein was loaded onto a GST-tagged resin column and filtered repeatedly to facilitate binding of the target protein to the GST tag. Unlabeled proteins (impurities) were washed away using Lysis buffer. To monitor the elution profile, 10 µL of flow-through was mixed with 50 µL of Bradford dye reagent. A blue color indicated high protein concentration in the eluate, while a colorless solution suggested effective removal of impurities. After thorough washing, the target protein was eluted using prepared elution buffer.

The conventional *in vitro* acyltransferase reaction system (10 μL) was prepared following previously described methods (Cao et al., 2024; Shen et al., 2021). The mixture consisted of 1 μL Tris-HCl (1 M, pH = 8), 0.5 μL substrate standard (100 μM), 0.5 μL acyl donor (500 μM), and 8 μL protein supernatant (500 ng). After incubation at 37 °C for 15 minutes, the reaction was terminated by adding 30 μL of HPLC-grade methanol. Proteins did not need to be removed. The sample was then centrifuged at 4 °C for 10 minutes at 13,000 rpm. Subsequently, 35 μL of the supernatant was transferred to a metabolic vial with an insert and stored at -20 °C until LC-MS/MS analysis.

### 2.5 Statistical analysis

Statistical analysis methods refer to published literature (Li et al., 2024). Differential metabolite analysis was performed by obtaining VIP values using OPLS-DA. Differentially accumulated metabolites (DAMs) were screened between the 06:00, 12:00, and 18:00 samples of castor roots, stems, and leaves using the criteria VIP > 1 and FoldChange ≥ 2 or FoldChange ≤ 0.5. The differentially expressed genes (DEGs) were mapped to the KEGG Orthology database and subsequently employed in the enrichment pathway analysis, following the method established (Minoru et al., 2008). A P-value threshold of less than 0.05 was utilized to identify significantly enriched KEGG pathways. For the visualization of phenolamides metabolite accumulation patterns, K-means clustering was implemented, leveraging the capabilities of the online metabolic clustering platform available at https://cloud.metware.cn (Gasch and Eisen, 2002). Molecular docking analysis was conducted using AlphaFold3 software for simulation analysis (Zhou et al., 2025). Input the protein sequence and small molecule SMILES into AlphaFold3, generate 5 protein complex structures using default parameters, select the model with the highest confidence, and visualize the evolution using PyMol. And the specific parameters for modeling are listed in **Supplementary Table 1**.

## 3 Results

### 3.1 Phenolamides analysis of different tissues of castor at different physiological clocks

**Figure 1:**
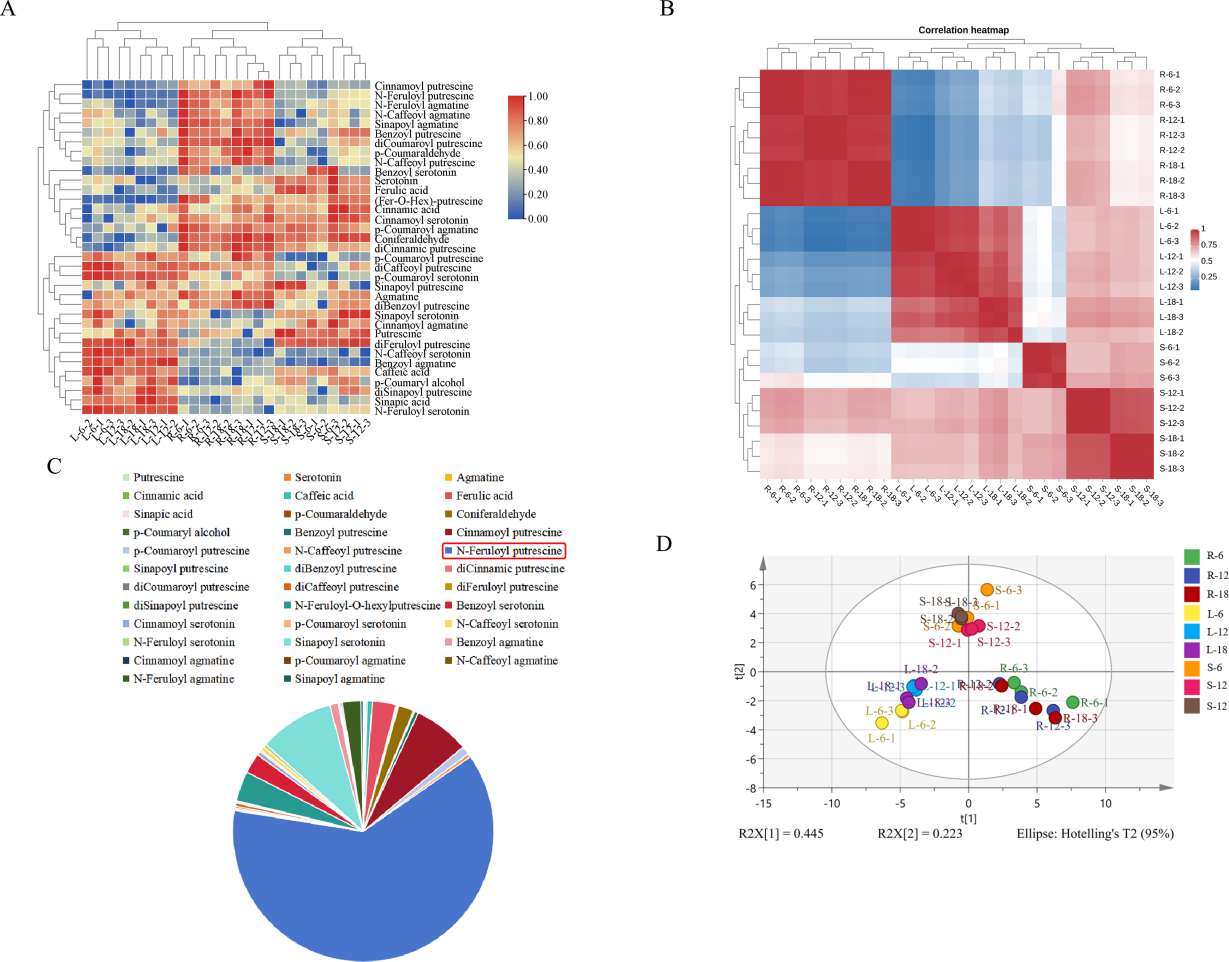
Analysis of phenolamides of castor. **(A)** Heat map analysis of the detected phenolamides metabolites of different tissues at different time points. High correlation in red and low correlation in blue. **(B)** Correlation clustering heatmap analysis of the detected phenolamides metabolites in different tissues at different time points. High correlation in red and low correlation in blue. **(C)** The statistical chart represents the proportion of all detected phenolamides metabolites. **(D)** PCA analysis of the detected phenolamides metabolites in different tissues at different time points.

Phenolamides were detected in castor root, stem, and leaf samples collected at 06:00, 12:00, and 18:00. A total of 35 phenolamides were identified (**Supplementary Table 2**). Analyses including heatmap, correlation, and PCA revealed significant differences in metabolite profiles among tissues and time points, particularly between root and leaf samples (**Figures 1A–D**). Correlation analysis indicated high intra-group reproducibility within root, stem, and leaf groups, suggesting strong experimental consistency, whereas low inter-group correlation highlighted distinct metabolic profiles across tissues. In stem tissue, phenolamides content was highest at 06:00 compared to 12:00 and 18:00 (**Figure 1B**). N-Feruloyl putrescine was the most abundant phenolamides (**Figure 1C**), with peak accumulation observed in root tissue at 06:00 and 18:00 (**Figure 1A**).

In addition, N-Feruloyl putrescine and cinnamoyl putrescine were found to accumulate abundantly in the roots. The level of N-Feruloyl putrescine was relatively high at 6:00, decreased after 12:00, and increased again after 18:00. In contrast, cinnamoyl putrescine exhibited the opposite pattern: its content in root tissues was lowest at 6:00, increased significantly after 12:00, and then declined sharply after 18:00.

In leaf tissues, three compounds (Sinapoyl serotonin, Benzoyl agmatine, and N-feruloyl putrescine) were accumulated to the greatest extent. Among them, Sinapoyl serotonin showed the highest level at 6:00, followed by a sequential decline at 12:00 and 18:00. Benzoyl agmatine exhibited the lowest levels at 6:00 and 18:00, but peaked at 12:00. In contrast, the content of N-feruloyl putrescine did not differ significantly among the three time points.

In stem tissues, metabolite accumulation differed markedly among the three time points examined. At 6:00, Benzoyl serotonin was the most abundant, followed by N-feruloyl putrescine and Sinapoyl serotonin. At 12:00, the level of N-feruloyl putrescine increased to its highest point, which was 2.5-fold higher than that at 6:00, and then returned to the 6:00 level by 18:00. A similar pattern was observed for Sinapoyl serotonin, whose content at 12:00 was 2.3-fold higher relative to 6:00, and likewise decreased to the 6:00 level by 18:00. In contrast, Benzoyl serotonin decreased sharply at 12:00, showing a 38-fold reduction, and then increased by approximately 2-fold at 18:00.

### 3.2 Phenolamides K-means analysis of different tissues of castor

**Figure 2:**
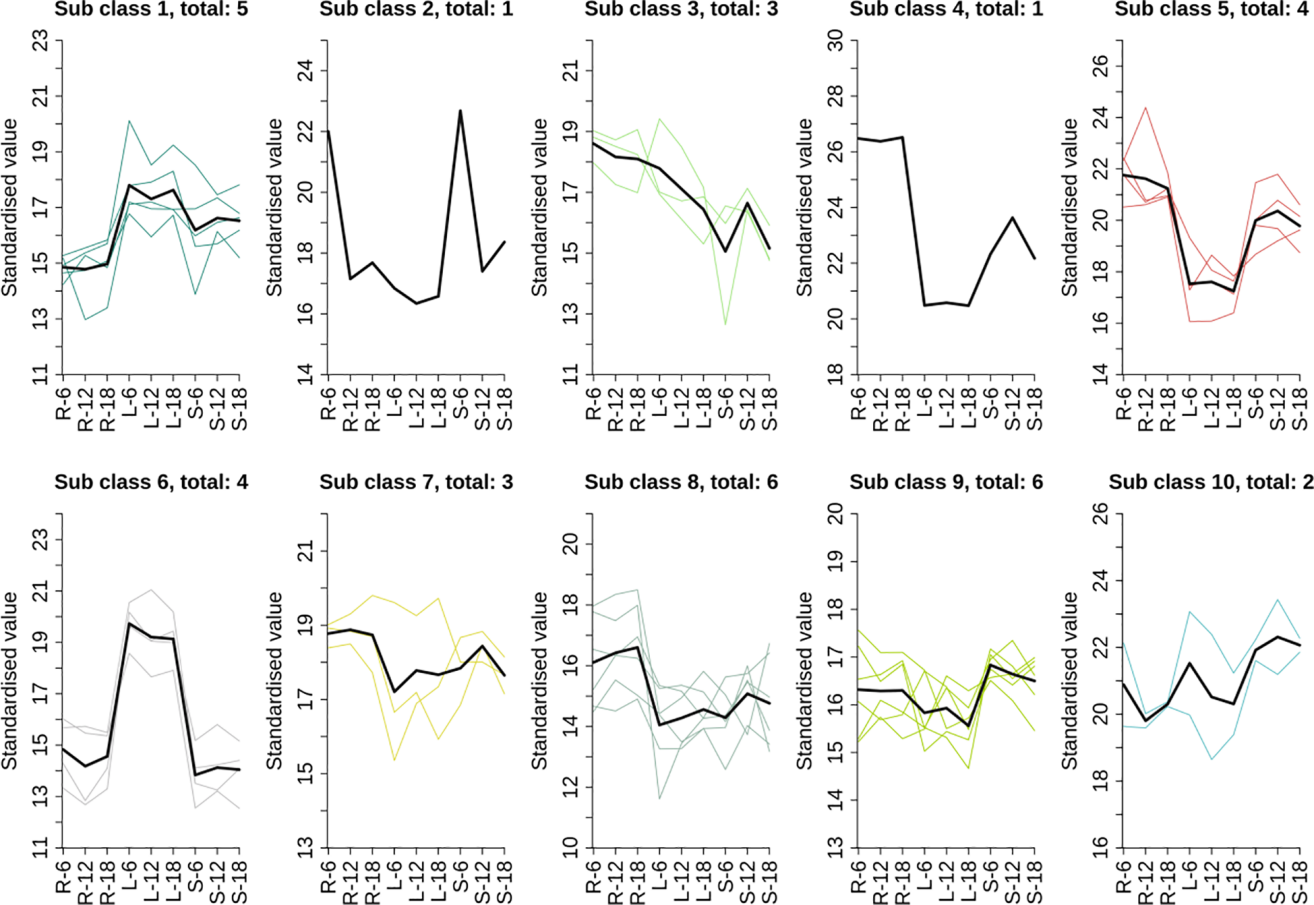
Phenolamide metabolomic K-means analysis of different tissues of castor. The horizontal axis represent three time points in different organizations, and the vertical axis represent the standardised values of relative metabolite content.

Using K-means clustering method, the phenolamides in root, stem, and leaf tissues at 06:00, 12:00, and 18:00 were divided into ten clusters, and the accumulation patterns of phenolamides in root, stem, and leaf tissues at 06:00, 12:00, and 18:00 were analyzed (**Figure 2**).

The increasing trend of phenolamides content in root tissues from 06:00 to 12:00 and then to 18:00 is clustered in clusters 1 and 8, with a total of 11 phenolamides. The decreasing trend is clustered in clusters 3, 5, and 9, with a total of 13 phenolamides. The trend of first decreasing and then increasing is clustered in clusters 2, 4, 6, and 10, with a total of 8 phenolamides. The clustering of the trend of first rising and then falling is in cluster 7, with a total of 3 phenolamides, indicating that the content of most phenolamides in root tissue is highest at 06:00.

The content of phenolamides in stem tissue from 06:00 to 12:00 and then to 18:00 showed a trend of first increasing and then decreasing in all clusters except for cluster 2. There were a total of 34 phenolamides, indicating that most of the phenolamides in stem tissue had the highest content at 12:00.

The decreasing trend of phenolamides content in leaf tissue from 06:00 to 12:00 and then to 18:00 is clustered in clusters 3, 6, and 10, with a total of 9 phenolamides. The trend of first decreasing and then increasing is clustered in clusters 1 and 2, with a total of 6 phenolamides. The trend of first increasing and then decreasing is clustered in clusters 4, 5, 7, 8, and 9, with a total of 20 phenolamides, indicating that most of the phenolamides in leaf tissue have the highest content at 12:00.

### 3.3 DAMs analysis of different tissues at different physiological clocks of castor

**Figure 3:**
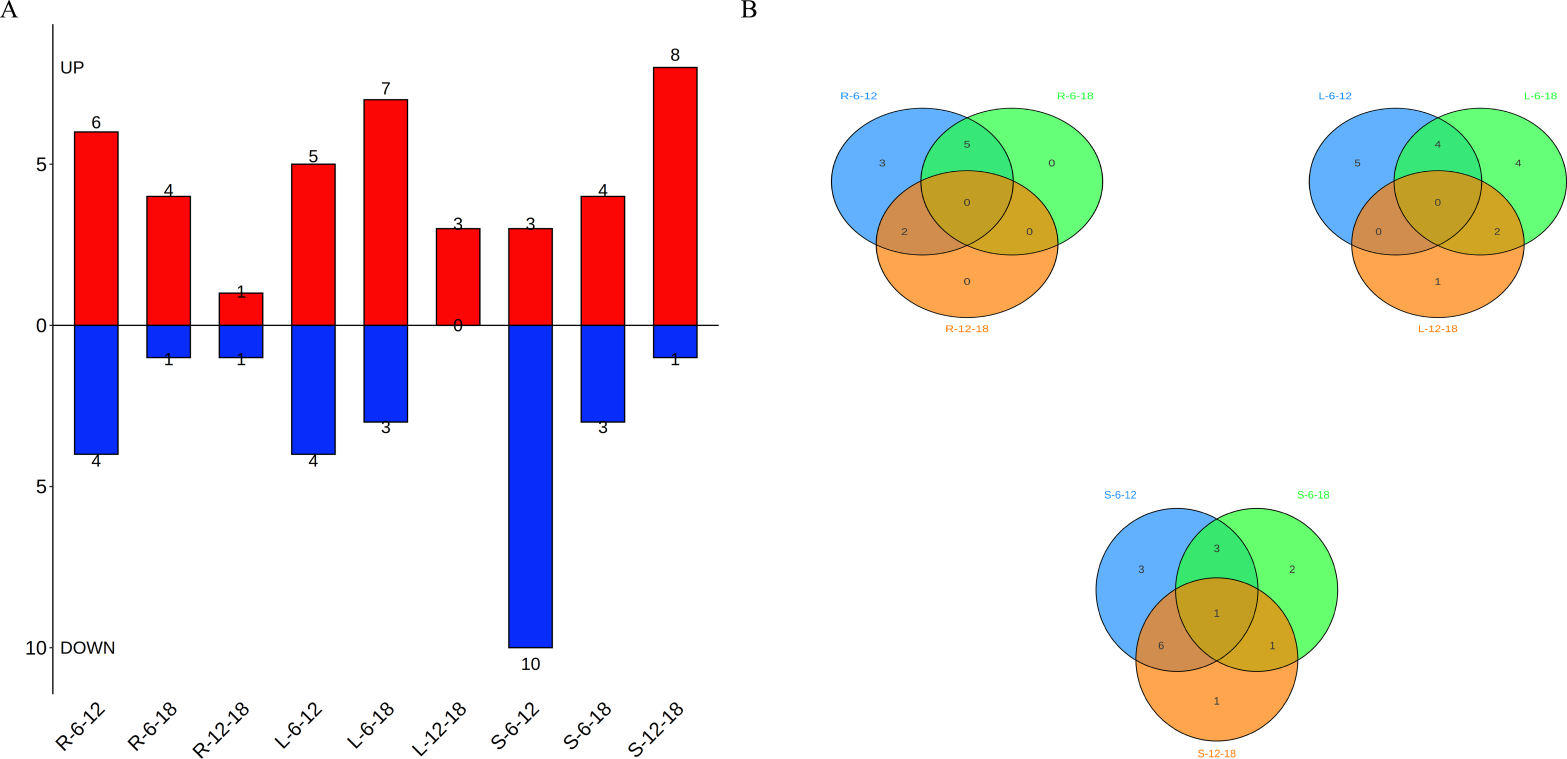
Analysis of DAMs of castor at different tissues. **(A)** Red represents upregulated phenolamides metabolites and blue represents downregulated phenolamides metabolites. **(B)** Venn diagram analysis of differentially expressed genes in different tissues at different time points.

A total of 35 phenolamides were identified in root, stem, and leaf tissues. The highest number of DAMs was observed in stem tissue, with 29 metabolites (15 upregulated, 14 downregulated), followed by leaf tissue with 22 DAMs (15 upregulated, 7 downregulated), and root tissue with 17 DAMs (11 upregulated, 6 downregulated) (**Figure 3A**).

Analysis of common DAMs across comparative groups revealed five shared metabolites between R-06:00 vs R-12:00 and R-06:00 vs R-18:00 in root tissue, whereas only two were common between R-06:00 vs R-12:00 and R-12:00 vs R-18:00. In leaf tissue, four common DAMs were identified between L-06:00 vs L-12:00 and L-06:00 vs L-18:00, with two shared between L-06:00 vs L-18:00 and L-12:00 vs L-18:00. For stem tissue, four common DAMs were detected between S-06:00 vs S-12:00 and S-06:00 vs S-18:00; two between S-06:00 vs S-18:00 and S-12:00 vs S-18:00; and seven between S-06:00 vs S-12:00 and S-12:00 vs S-18:00 (**Figure 3B**).

### 3.4 Transcriptome analysis of different tissues of castor at different physiological clocks

Following RNA-Seq-based transcriptome profiling of castor root, stem, and leaf tissues, all identified genes were subjected to KEGG pathway analysis (**Supplementary Figure 2**) (Li et al., 2024). Significant enrichment of the phenylpropanoid biosynthesis pathway was observed among downregulated genes in leaf tissue at 6:00 vs 18:00, and 12:00 vs 18:00, respectively. (**Supplementary Figures 2A, B**). In roots, significant enrichment of phenylpropanoid biosynthesis and enrichment of tryptophan metabolism were observed in the downregulated genes at 6:00 vs 18:00. (**Supplementary Figures 2C, D**). Conversely, enrichment in stem tissue was minimal, with only the phenylpropanoid biosynthesis being slightly enriched among genes upregulated at 06:00 vs 12:00 (**Supplementary Figures 2E, F**). Phenolamides biosynthesis involves two key processes: hydroxycinnamoyl-CoA synthesis through the phenylpropanoid biosynthesis pathway and polyamine formation via tryptophan metabolism pathway. Enzymatic modification of these intermediates by hydroxycinnamoyl transferase then yields phenolamides (Peng et al., 2016b; Xu et al., 2022). Therefore, DEGs were involved in the phenolamides synthesis in different tissues of castor.

**Figure 4:**
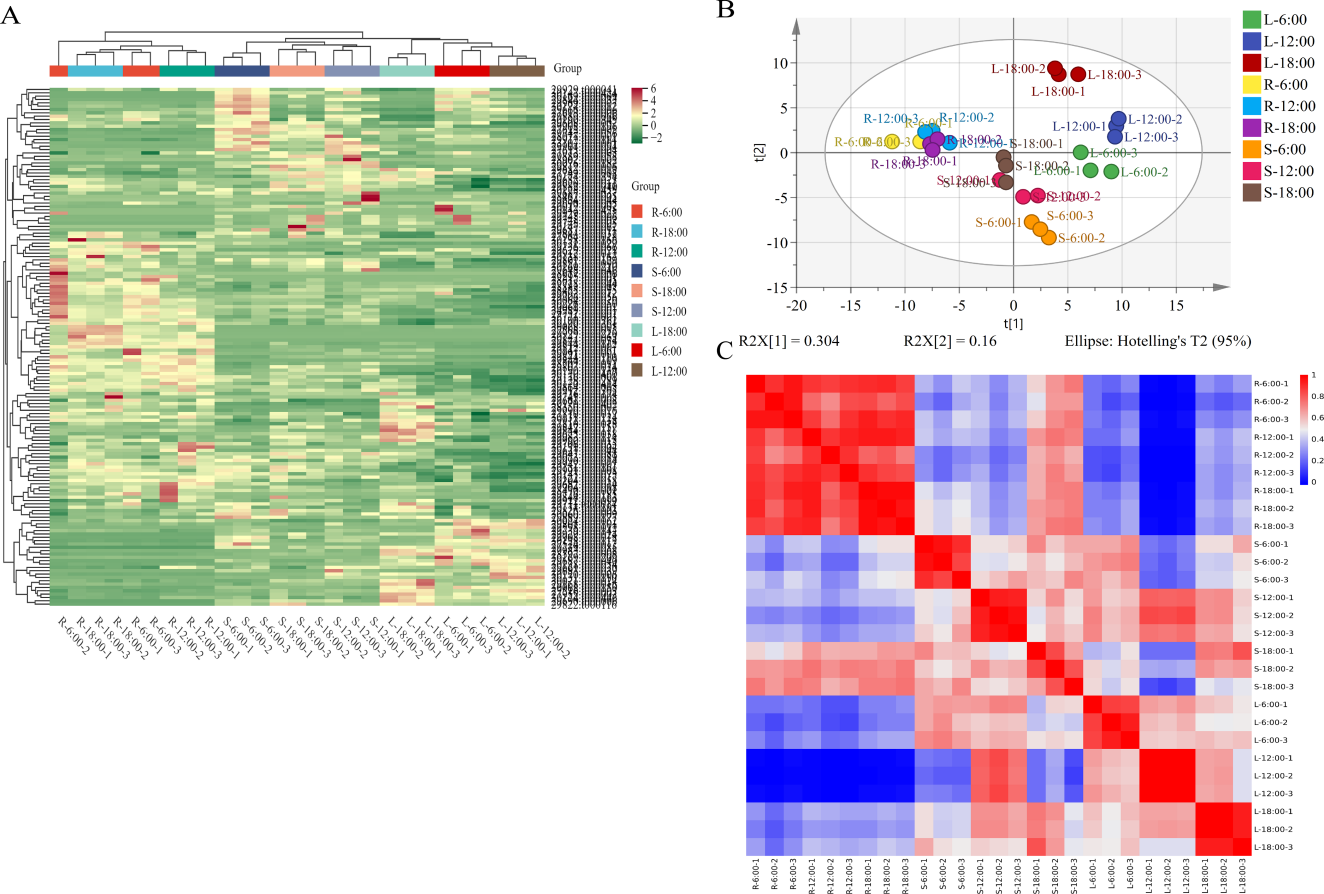
Cluster analysis of castor acyltransferase genes. **(A)** Heat map analysis of acyltransferase genes of different tissues at different time points. High correlation in red and low correlation in green. **(B)** PCA analysis of acyltransferase genes in different tissues at different time points. **(C)** Correlation clustering heatmap analysis of acyltransferase genes in different tissues at different time points. High correlation in red and low correlation in green.

After analyzing the gene annotation of castor tissues across multiple time points, 156 acyltransferase genes were identified (**Supplementary Table 4**). These genes were subsequently analyzed using correlation heatmaps, cluster heatmaps, and principal component analysis (PCA). The results indicated significant differential expression of these genes among root, stem, and leaf tissues. Notably, pronounced expression variations were observed across tissues at 06:00, 12:00, and 18:00, while high reproducibility was evident within the same tissue at identical time points (**Figures 4A–C**).

**Figure 5:**
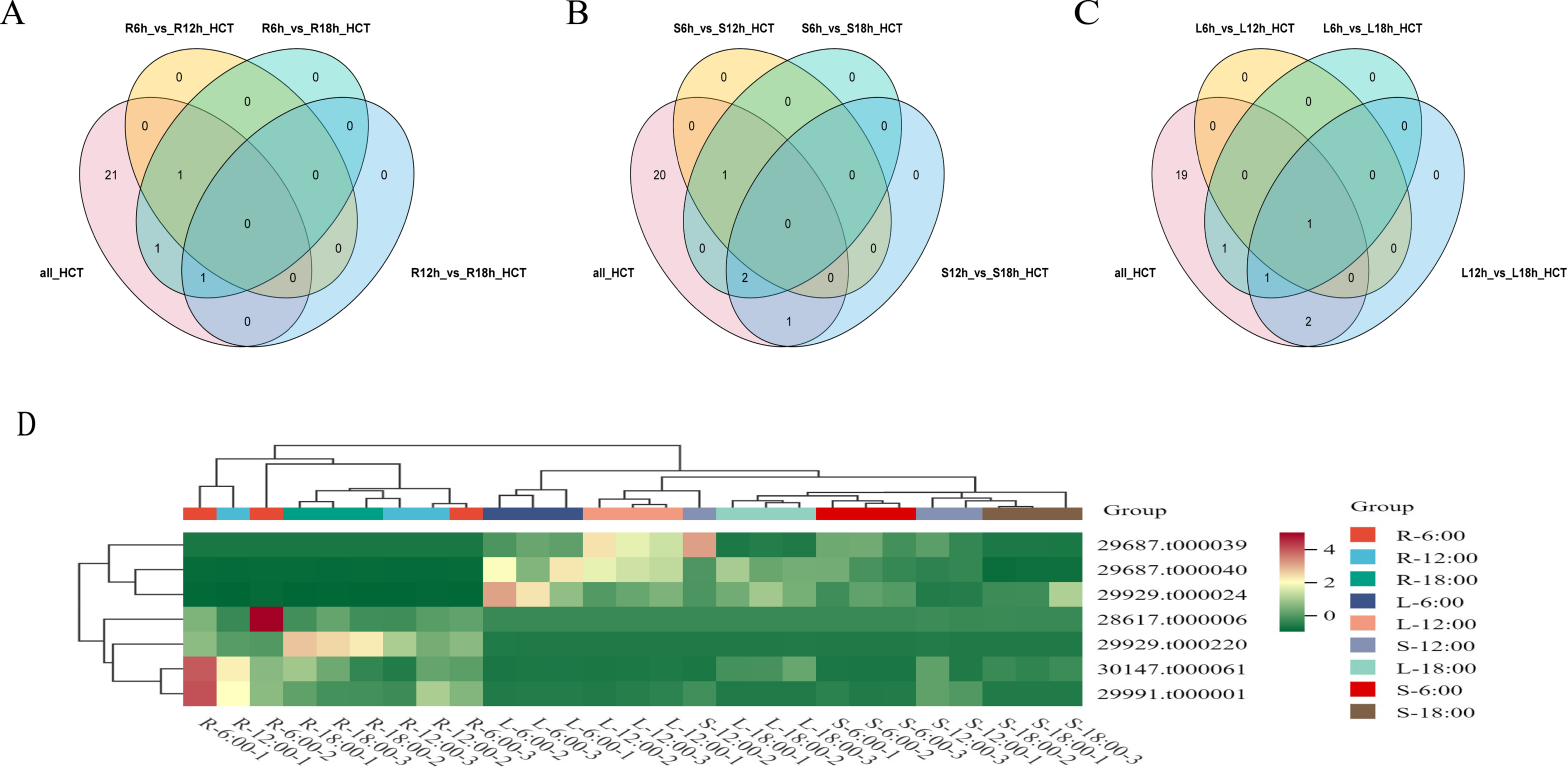
Venn diagram analysis and expression profile analysis of HCTs. **(A-C)** Venn diagram analysis of HCTs genes in root, stem and leaf tissues at different time points. **(D)** Expression profile analysis of 7 HCTs genes in different tissues at different time points. Red indicates high correlation, green indicates low correlation.

Among the 156 acyltransferase genes identified, 24 acyltransferase genes were annotated as HCT (**Supplementary Table 3**). Venn diagram analysis revealed 3 DEGs of HCT in root tissues across time points, followed by 4 DEGs of HCT in stems and 5 DEGs of HCT in leaves at 06:00, 12:00 and 18:00. Additionally, 7 DEGs of HCT were common to all three tissues (**Figures 5A–C**, **Supplementary Table 5**). And showed the expression profiles of 7 common DEGs of HCT in castor root, stem and leaf tissues at 06:00, 12:00 and 18:00 (**Figure 5D**).

### 3.5 Correlation analysis of differential metabolites and differential genes in different tissues of castor at different physiological clocks

**Figure 6:**
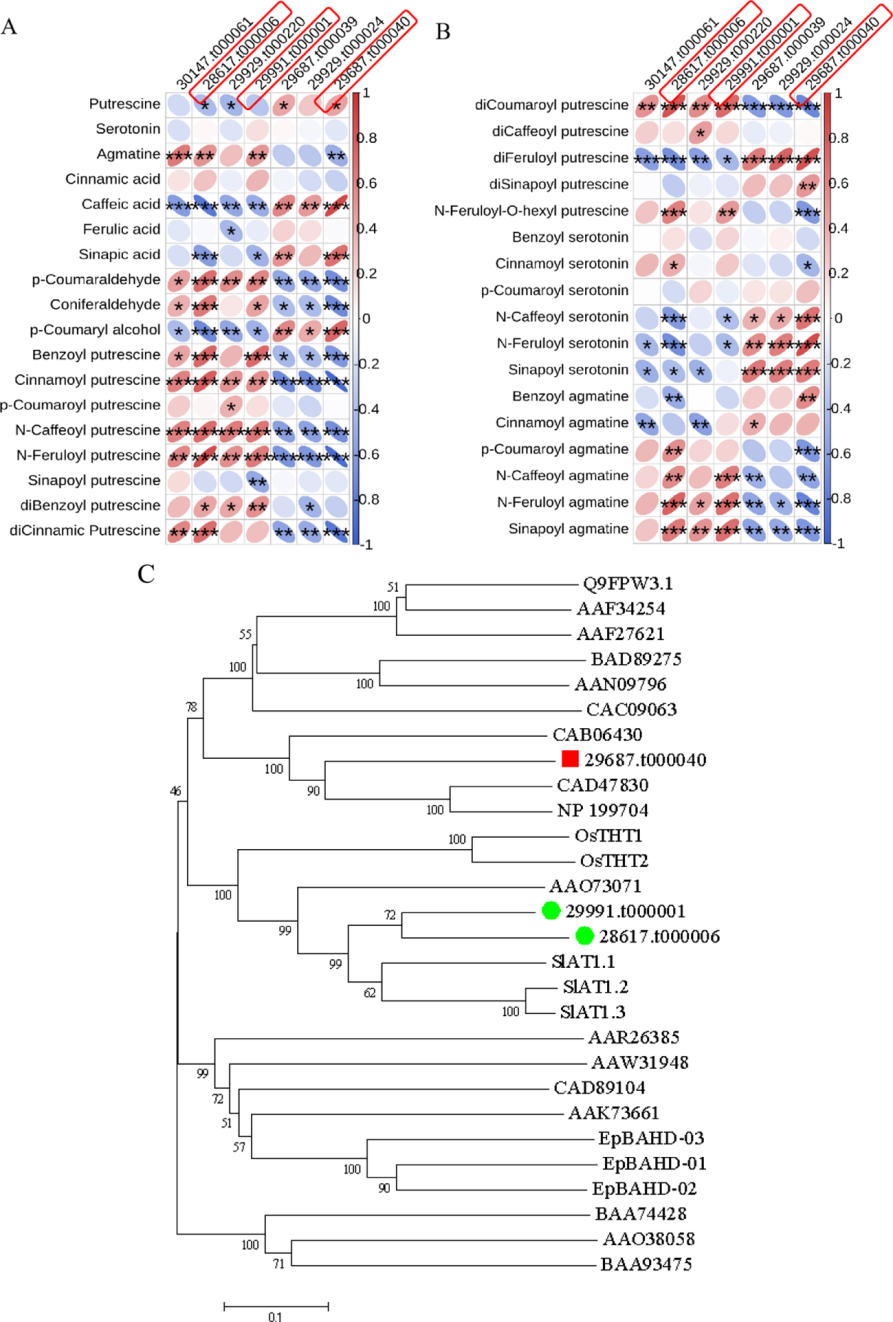
Multi omics correlation analysis and evolutionary tree analysis of candidate genes. **(A, B)** Correlation analysis between 35 phenolamides metabolites and 7 HCTs. High correlation in red and low correlation in blue. **(C)** Phylogenetic tree analysis of BADH family genes in different species. The asterisk represents correlation, and the more asterisks there are, the higher the correlation.

A total of 35 phenolamides and 7 differentially expressed HCT genes were identified in this study. These were further subjected to integrated multi-omics correlation analysis (with |r| ≥ 0.7) (**Figures 6A, B**). The results demonstrated a strong positive correlation between gene *29687.t000040* and both caffeic acid (r = 0.89) and N-Feruloyl serotonin (r = 0.87). Similarly, gene *28617.t000006* showed high correlation with N-Feruloyl putrescine (r = 0.86) and dicoumaroyl putrescine (r = 0.84). Additionally, gene *29991.t000001* was also highly correlated with multiple phenolamides, including benzoyl putrescine and dicoumaroyl putrescine.

*28617.t000006* and *29991.t000001* exhibited a co-expression pattern, with peak expression predominantly observed in root tissue at 06:00 (**Figure 5D**). In contrast, gene *29687.t000040* displayed a distinct expression profile, showing highest expression in leaf tissue at the same time point (**Figure 5D**). Phylogenetic analysis including HCTs from *Solanum lycopersicum*, *Oryza sativa*, and several *Euphorbiaceae* species revealed that *28617.t000006* and *29991.t000001* clustered within the same clade, sharing 70.43% homology. However, neither gene clustered with *29687.t000040*, with which they exhibited low sequence similarity 32.33% and 32.08%, respectively (**Figure 6C**). Therefore, it is speculated that *29687.t000040*, *28617.t000006* and *29991.t000001* have different evolutions in terms of gene function. Further *in vitro* enzymatic assays will be conducted to validate their roles in the acylation modification of polyamines.

### 3.6 Functional characterization of candidate genes for biosynthesis of castor

**Figure 7:**
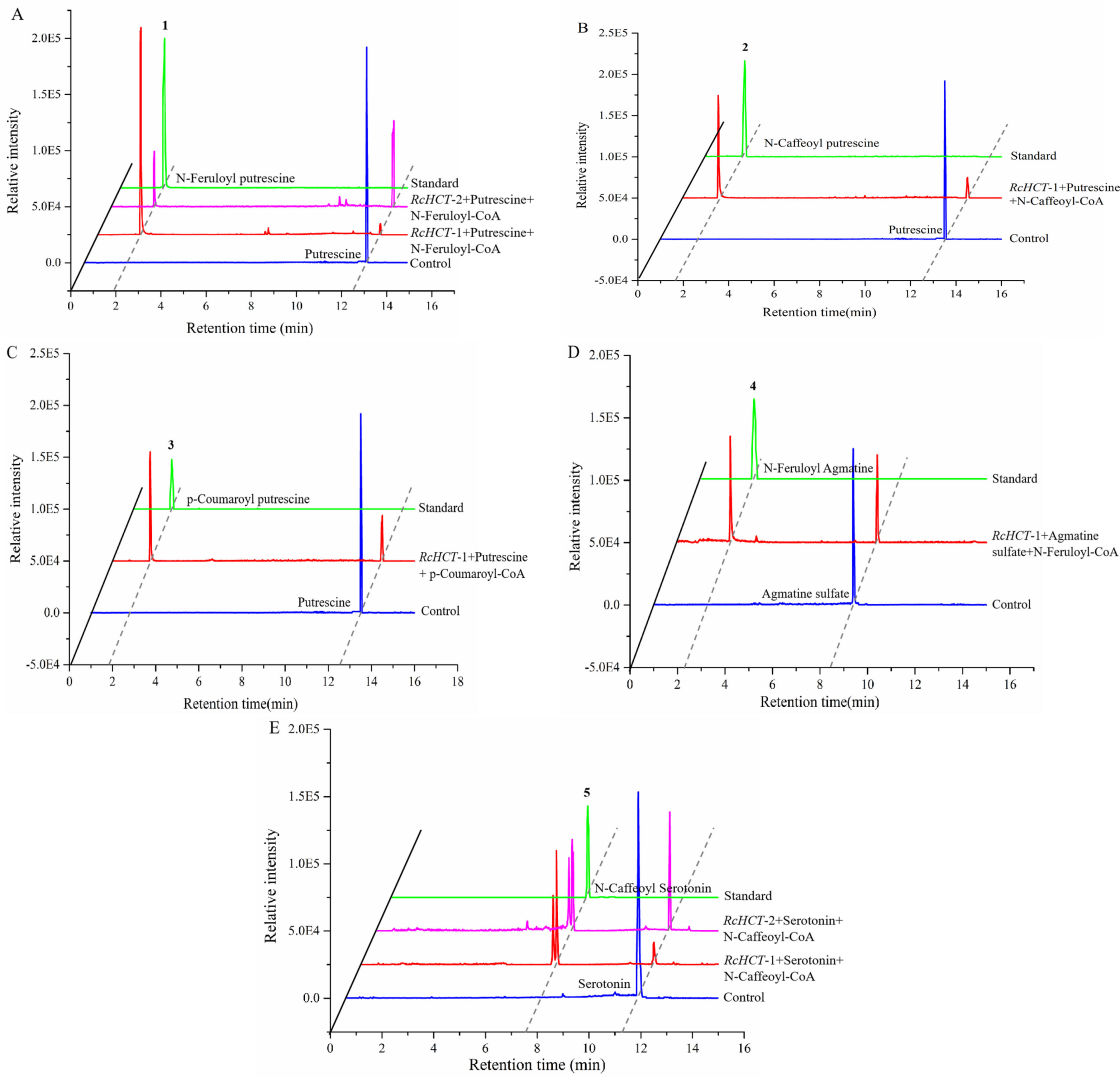
Functional analysis of *in vitro* acylation modification of *RcHCT1* and *RcHCT2.* **(A)** The acylation modification of *RcHCT1* and *RcHCT2* using putrescine as substrate and N-Feruloyl-CoA as donor resulted in product 1 being N-Feruloyl putrescine. **(B)** The acylation modification of *RcHCT1* using putrescine as substrate and N-Caffeoyl-CoA as donor resulted in product 2 being N-Caffeoyl putrescine. **(C)** The acylation modification of *RcHCT1* using putrescine as substrate and p-Coumaroyl-CoA as donor resulted in product 3 being p-Coumaroyl putrescine. **(D)** The acylation modification of *RcHCT1* using agmatine as substrate and N-Feruloyl-CoA as donor resulted in product 4 being N-Feruloyl agmatine. **(E)** The acylation modification of *RcHCT1* and *RcHCT2* using serotonin as substrate and N-Caffeoyl-CoA as donor resulted in product 5 being N-Caffeoyl serotonin. The secondary mass spectrometry fragment information of the products were shown in **Supplementary Figure 4**.

The functions of three candidate genes *28617.t000006*, *29991.t000001* and *29687.t000040* were investigated through prokaryotic expression systems and *in vitro* enzymatic assays using polyamine substrates. Enzyme activity was assessed with putrescine, serotonin, and agmatine sulfate as acyl acceptors, and N-Feruloyl-CoA, N-Caffeoyl-CoA, and p-Coumaroyl-CoA as acyl donors. Corresponding substrates and reaction products were detected via LC-MS/MS. However, *in vitro* enzymatic results confirmed acyltransferase activity only for *28617.t000006* and *29991.t000001* (**Figure 7**). Specifically, *29991.t000001* catalyzed the acyl modification of putrescine using N-Feruloyl-CoA, N-Caffeoyl-CoA, and p-Coumaroyl-CoA, yielding produce 1, 2, 3:N-Feruloyl putrescine, N-Caffeoyl putrescine, and p-Coumaroyl putrescine, respectively (**Figures 7A–C**). Using putrescine as a substrate and N-Feruloyl-CoA as the acyl donor, *28617.t000006* also catalyzed the formation of N-Feruloyl putrescine (**Figure 7A**). Meanwhile, *29991.t000001* utilized agmatine as a substrate and N-Feruloyl-CoA as the donor to produce 4:N-Feruloyl agmatine (**Figure 7D**). Additionally, both 29991.t000001 and 28617.t000006 were capable of utilizing serotonin as a substrate and N-Caffeoyl-CoA as the acyl donor to synthesize produce **5**:N-Caffeoyl serotonin (**Figure 7E**). Therefore, *29991.t000001* (*LOC8267026*) and *28617.t000006* (*LOC8266728*) were identified as multifunctional hydroxycinnamoyl transferases that catalyze the formation of amide metabolites from various polyamine substrates. Both genes belong to the BAHD acyltransferase family and have been designated as *RcHCT1* (*29991.t000001*) and *RcHCT2* (*28617.t000006*), respectively.

In summary, *RcHCT1* was capable of utilizing putrescine, serotonin, and agmatine as acyl acceptors with N-Feruloyl-CoA, N-Caffeoyl-CoA, and p-Coumaroyl-CoA as donors to catalyze the formation of N-Feruloyl putrescine, N-Caffeoyl putrescine, p-Coumaroyl putrescine, N-Caffeoyl serotonin, and N-Feruloyl agmatine, respectively (**Supplementary Figure 5**). In contrast, *RcHCT2* only accepts putrescine and serotonin as substrates, using N-Feruloyl-CoA and N-Caffeoyl-CoA to produce N-Feruloyl putrescine and N-Caffeoyl serotonin, respectively (**Supplementary Figure 5**). Both *RcHCT1* and *RcHCT2* represent previously unreported members of the BAHD acyltransferase family identified in *Ricinus communis* L.

### 3.7 Subcellular localization of *RcHCT1* and *RcHCT2*

**Figure 8:**
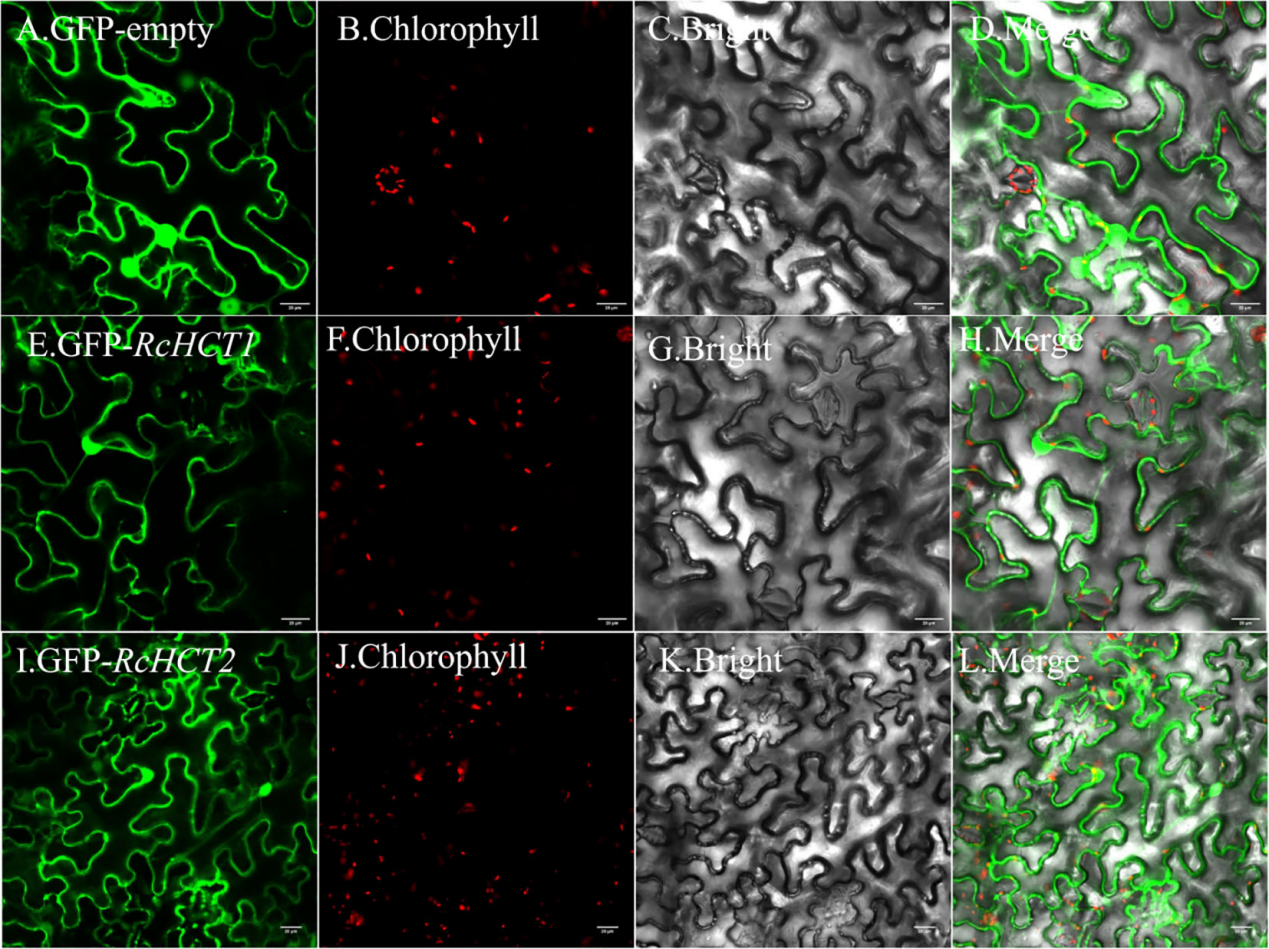
Subcellular localization of *RcHCT1* and *RcHCT2* in the epidermal cells of Ncotianaberhaniana laves. **(A)** 35S::eGFP-empty. **(B, F, J)** Chlorophyll spontaneous fluorescence. **(C, G, K)** The bright feld view. **(D, H, L)** The overlayed signal of the eGFP, chlorophyll and the bright feld view. **(E)** 35S:: *RcHCT1*-eGFP. **(I)** 35S:: *RcHCT2*-eGFP. The scale bar represents 20 µm.

Subcellular localization analysis of *RcHCT1* and *RcHCT2* revealed that the experimental group exhibited the same localization pattern as the empty vector control, with both proteins localized to both the nucleus and the cytoplasm (**Figure 8**).

### 3.8 Molecular docking simulation analysis of *RcHCT1* and *RcHCT2* with substrates

**Figure 9:**
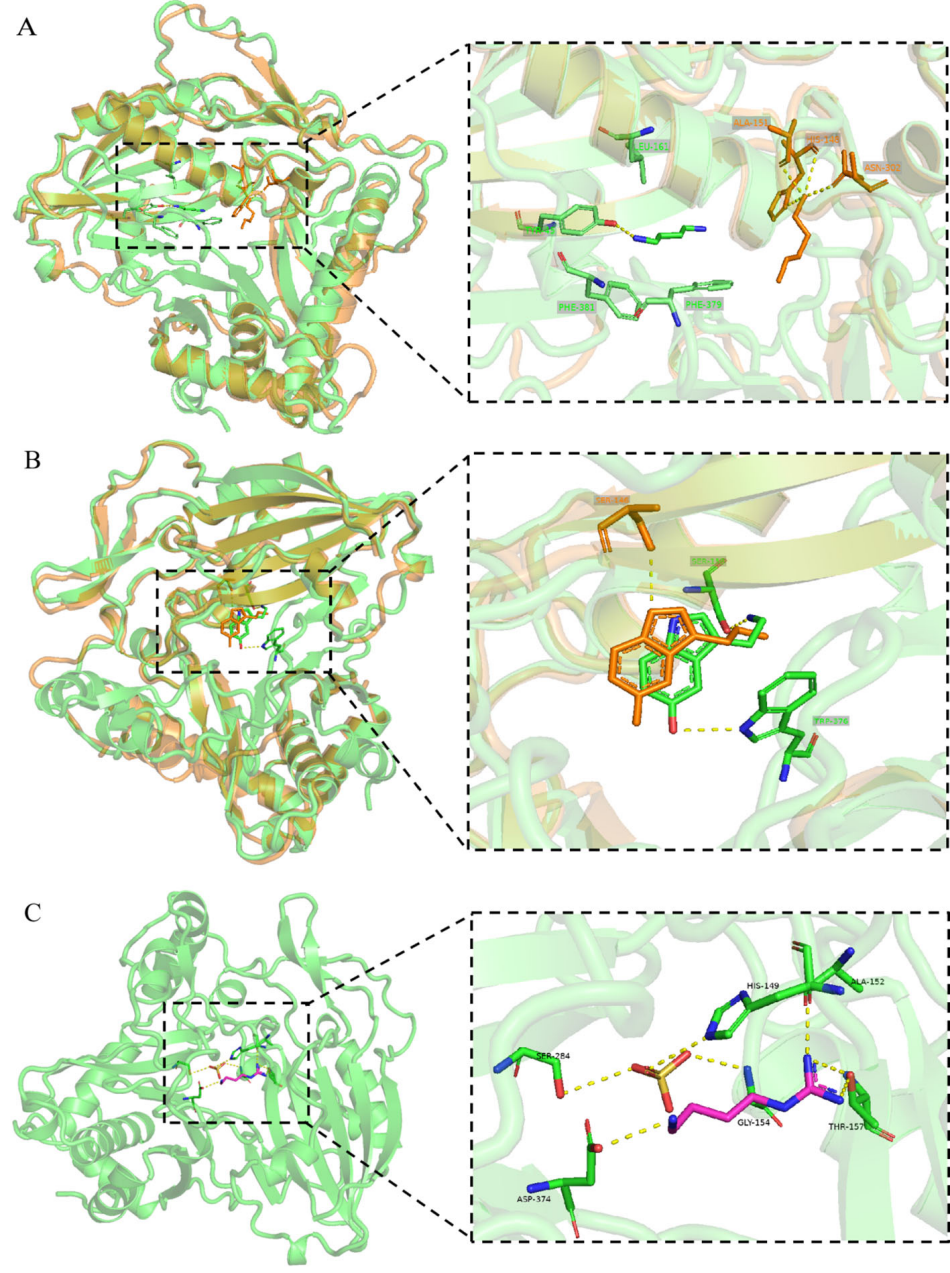
Molecular docking analysis of *RcHCT1*and *RcHCT2* with substrates. **(A)** Green represents the molecular docking between *RcHCT1* and putrescine, while orange represents the molecular docking between *RcHCT2* and putrescine, yellow represents hydrogen bonds. **(B)** Green represents the molecular docking between *RcHCT1* and serotonin, while orange represents the molecular docking between *RcHCT2* and serotonin, yellow represents hydrogen bonds. **(C)** Green represents the molecular docking between *RcHCT1* and agmatine sulfate, yellow represents hydrogen bonds.

Based on AlphaFold3-predicted structures of *RcHCT1* and *RcHCT2*, molecular docking simulations were performed to characterize substrate binding. With putrescine as a substrate, hydrogen bonds were formed with TYR45 in *RcHCT1*, and with HIS148, ALA151, and ASN302 in *RcHCT2* (**Figure 9A**). In *RcHCT1*, three peripheral residues (LEU161, PHE379, and PHE381) were located near the active pocket but did not directly interact with putrescine. The docking ranking score was higher for the *RcHCT1*-putrescine complex (0.91) than for *RcHCT2* (0.86). Furthermore, both the Predicted TM-score (PTM) and Interface PTM (IPTM) were greater for *RcHCT1* (PTM = 0.92, IPTM = 0.91) than for *RcHCT2* (PTM = 0.70, IPTM = 0.84) (**Supplementary Table 1**), collectively indicating higher modeling confidence for *RcHCT1*.

When docked with serotonin, hydrogen bonds were formed with TRP376 and SER158 in *RcHCT1*, and with SER146 in *RcHCT2* (**Figure 9B**). The global model quality, as reflected by the PTM score, was significantly higher for *RcHCT1* (0.92) than for *RcHCT2* (0.76). Although *RcHCT2* exhibited marginally higher IPTM and docking scores (0.87 and 0.89, respectively) compared to *RcHCT1* (0.83 and 0.85) (**Supplementary Table 1**), the marked superiority of *RcHCT1* in global fold accuracy suggests that its complex with serotonin possesses higher overall reliability. The slightly better interface metrics for *RcHCT2* may indicate a more optimized local binding mode for serotonin.

Regarding agmatine sulfate, its agmatine moiety formed hydrogen bonds with ASP374, THR157, and ALA152 in *RcHCT1* (**Figure 9C**). This complex yielded a docking score of 0.85, with a PTM of 0.91 and an IPTM of 0.83 (**Supplementary Table 1**).

## 4 Discussion

### 4.1 Accumulation patterns of phenolamides and candidate genes

Metabolomic profiling of castor root, stem, and leaf tissues at 06:00, 12:00, and 18:00 identified 35 phenolamides, the levels of which exhibited substantial variation across tissues and time points (**Figure 1**). K-Means clustering analysis revealed that most phenolamides reached their highest abundance in root tissue at 06:00 (**Figure 2**). This accumulation pattern may be associated with the absence of sunlight, consistent with previous reports indicating that the total flavonoid (TFC) and the total phenolic (TPC) contents are also highest in root tissues at R-06:00 under minimal light conditions (Li et al., 2024). In contrast, stem and leaf tissues showed peak accumulation of most phenolamides at 12:00 (**Supplementary Table 2**). The metabolic pattern in leaves may correlate with solar radiation, as supported by findings that TFC and TPC levels are highest at L-12:00 under intense midday sunlight (Li et al., 2024). Based on the results, it can be preliminarily inferred that at 6:00 before the sun comes out, castor does not need to resist the stress of UV rays from the sun. At this time, a large amount of phenolamides were stored in the roots to promote their salt or drought tolerance and other stress resistance effects. After 12:00, strong UV radiation causes stress on castor leaves, and phenolamides from the roots were transported to the leaves and stem to resist UV stress. Until 18:00, when the intensity of UV stress decreased, phenolamides in leaf and stem tissues returned to root tissue to exert their functions. Overall, the spatiotemporal accumulation patterns of phenolamides align closely with those of flavonoids across tissues and time points.

Among the 35 phenolamides identified, N-Feruloyl putrescine was the most abundant. Its signal intensity was highest in root tissue (9.15 × 10^7^), compared to stem (1.48 × 10^6^) and leaf (7.81 × 10^6^) tissues, indicating predominant accumulation in roots (**Supplementary Table 2**). Furthermore, within root tissue, N-Feruloyl putrescine levels were elevated at 06:00 and 18:00, and the lowest at 12:00. In contrast, leaf tissue showed lower abundance at 06:00 and 18:00, but higher accumulation at 12:00 (**Supplementary Table 2**). Transcriptome analysis revealed that the expression levels of *RcHCT1* (*29991.t000001*) and *RcHCT2* (*28617.t000006*) were also highest in root tissues at 06:00 and lower at 12:00, consistent with the metabolite accumulation pattern (**Supplementary Tables 3**, **5**). KEGG pathway analysis revealed that genes downregulated in root tissues from 06:00 to 18:00 were significantly enriched in tryptophan and phenylpropanoid biosynthesis, with the latter showing particularly strong enrichment. In contrast, these pathways were not enriched in leaf or stem tissues (**Supplementary Figure 2**). This tissue-specific pattern reinforces the consistency between gene expression and metabolite accumulation in roots.

The resistance of rice to UV-B stress can be enhanced through UV-B induced biosynthesis of phenolamides, a process documented to involve the accumulation of these metabolites and the upregulation of associated gene expression (Cao et al., 2024; Dong et al., 2015; Peng et al., 2016b). Subsequent experiments will involve UV-B radiation treatment of castor plants to monitor dynamic changes in phenolamides levels and expression of its biosynthetic genes under induced conditions.

### 4.2 Functional analysis of biosynthesis genes of phenolamides

In this study, following established methods (Xu et al., 2023, Xu et al., 2021), integrated metabolomic and transcriptomic correlation analysis identified three candidate hydroxycinnamoyl transferase genes *RcHCT1*, *RcHCT2*, and *29687.t000040* in castor exhibiting strong correlations with metabolite profiles. Phylogenetic analysis of protein sequences revealed that *29687.t000040* shares low homology with *RcHCT1* and *RcHCT2* and does not cluster on the same clade (**Figure 6C**). Therefore, it is speculated that *RcHCT1*, *RcHCT2*, and *29687.t000040* have different evolutions in terms of gene function. Interestingly, subsequent *in vitro* functional assays confirmed that only *RcHCT1* and *RcHCT2* catalyze the acylation of polyamines, whereas *29687.t000040* showed no detectable activity in this regard. It is plausible that *29687.t000040* exhibits limited substrate adaptability toward putrescine, serotonin, and agmatine sulfate, and that its optimal modification substrates remain unidentified.

*In vitro* enzymatic assays demonstrated that *RcHCT1* displays broad substrate promiscuity, accepting multiple polyamine substrates as acyl acceptors and various acyl-CoA donors to generate diverse phenolamides. This functional versatility suggests a robust catalytic capacity of *RcHCT1* in phenolamides biosynthesis, consistent with the previously described acceptor flexibility of the *ElBAHD16* gene family in *Euphorbiaceae* (Zhao et al., 2025). Moreover, the mechanism by which *RcHCT1* catalyzes polyamine acylation aligns with reported functions of phenolamides hydroxycinnamoyl transferase genes in rice and tomato (Cao et al., 2024; Dong et al., 2015; Peng et al., 2016b).

**Figure 10:**
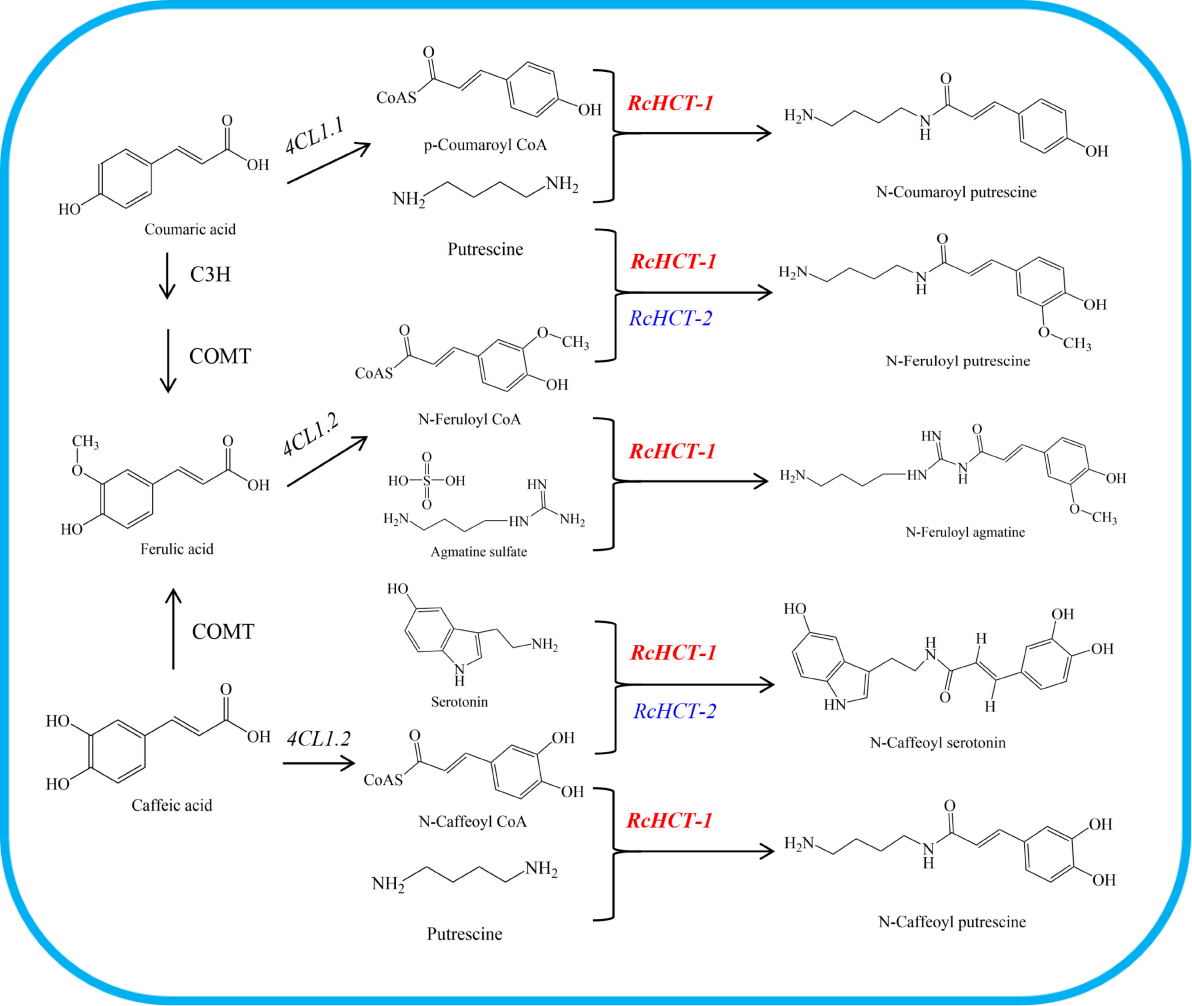
Analysis of the biosynthetic pathway of phenolamides of castor. (4CL1.1, 4CL1.2 respectively represent the reported genes: 4-coumarate-CoA ligase genes).

However, compared to *RcHCT1*, *RcHCT2* exhibits narrower substrate specificity toward polyamines. Both *RcHCT1* and *RcHCT2* belong to the BAHD acyltransferase family and represent the first reported hydroxycinnamoyl transferases involved in phenolamide biosynthesis in *Ricinus communis* L.(**Figure 10**). Although BAHD acyltransferases have been previously identified in *Euphorbiaceae*, those reported to date are primarily associated with the modification of diterpenes for macrocyclic diterpene synthesis (Schotte et al., 2025; Zhao et al., 2025), rather than hydroxycinnamoyl transferases specific for phenolamide formation. Owing to the limited availability of authentic standards, it was not feasible to compare the catalytic efficiencies (*Kcat*/*Km*) of *RcHCT1* and *RcHCT2*, thereby precluding a quantitative assessment of their relative catalytic activities.

Subcellular localization analysis revealed that both RcHCT1 and RcHCT2 were distributed in the nucleus and cytoplasm. This dual localization pattern suggests that they may function in multiple cellular compartments. While their cytoplasmic forms likely catalyze the bulk acylation of amine derivatives (e.g., putrescine, serotonin) to form defensive hydroxycinnamic acid amides, their nuclear pools might be involved in the acylation of nuclear proteins or histones, thereby linking secondary metabolism to epigenetic regulation.

Molecular docking simulations results indicate that the catalytic sites within the active pockets are crucial for the enzymes’ function, and variations in these residues may account for the catalytic differences between *RcHCT1* and *RcHCT2*. Although the two proteins were closely related, alterations to the active site can affect its spatial configuration and potentially impact catalytic function (Zhou et al., 2025). Molecular docking further revealed a higher binding affinity of *RcHCT1* than *RcHCT2* for the putrescine and serotonin. Interestingly, the molecular docking predictions were validated by our *in vitro* enzyme activity assays. Specifically, the assays showed that *RcHCT1* consumed greater quantities of putrescine and serotonin and yielded more product than *RcHCT2* (**Figures 7A, E**), which is consistent with its higher predicted binding affinity. The primary focus of this study was the preliminary validation of the gene functions of *RcHCT1* than *RcHCT2*. Unfortunately, this work did not involve in-depth site-directed mutagenesis of key amino acid residues to enhance the enzymatic activity of the recombinant proteins.

## 5 Conclusion

In summary, N-Feruloyl putrescine is the most abundant phenolamides, with its highest accumulation observed in root tissue at 06:00. Through integrated metabolomic and transcriptomic analysis, two BAHD family HCT genes, *RcHCT1* and *RcHCT2*, were first identified and functionally validated in castor. Both genes exhibited peak expression in root tissue at 06:00. Among them, *RcHCT1* demonstrated broad substrate adaptability, catalyzing the formation of N-Feruloyl putrescine, N-Caffeoyl putrescine, p-Coumaroyl putrescine, N-Caffeoyl serotonin, and N-Feruloyl agmatine from putrescine, serotonin, and agmatine sulfate. In contrast, *RcHCT2* showed narrower substrate specificity, utilizing only putrescine and serotonin to produce N-Feruloyl putrescine and N-Caffeoyl serotonin, respectively. And subcellular localization of *RcHCT1* and *RcHCT2* indicates that both proteins are localized to both the nucleus and the cytoplasm. Furthermore, molecular docking was employed to simulate the active sites and identify the key amino acid residues within *RcHCT1* and *RcHCT2* that interact with their respective substrates. These findings provide a theoretical foundation for the future development and utilization of bioactive phenolamide compounds in castor.

## Data Availability

The original contributions presented in the study are included in the article/**Supplementary Material**. Further inquiries can be directed to the corresponding authors.
