# Supplementary material for: Analysis of biosynthetic genes of phenolamides in Ricinus communis L. based on metabolomics and transcriptomes
Source: Front Plant Sci. 2026 May 25;17:1836215. doi: 10.3389/fpls.2026.1836215 (PMC13243259; doi:10.3389/fpls.2026.1836215)
Supplement: Supplementary file 2 [file Table2.docx]

**Analysis of biosynthetic genes of phenolamides metabolites in *Ricinus communis* L. based on metabolomics and transcriptomes**

Li Hua^a 1^, Huang Sishu^b 1^, Zhang Xiaojia^a 1^, Xu Congping^c^, Li Saiwen^a^, Zhan Chuansong^b^ ^*^, Luo Yanping^a^ ^*^

^a^ School of Tropical Agriculture and Forestry, School of Ecology, Hainan University, Haikou 570228, China

^b^ Yazhouwan National Laboratory (YNL), Sanya 572025, China

^C^ School of Life Science and Technology, Wuhan Polytechnic University, Wuhan 430023, China.

^1^ These authors have contributed equally to this work

^*^ Corresponding authors:E-mail addresses: [yanpluo2012@hainanu.edu.cn](mailto:jie.luo@hainanu.edu.cn;) (Luo Y.P.) and chuansongzhan01@foxmail.com(Zhan C.S.).

**Supplementary Figure S1-S3**


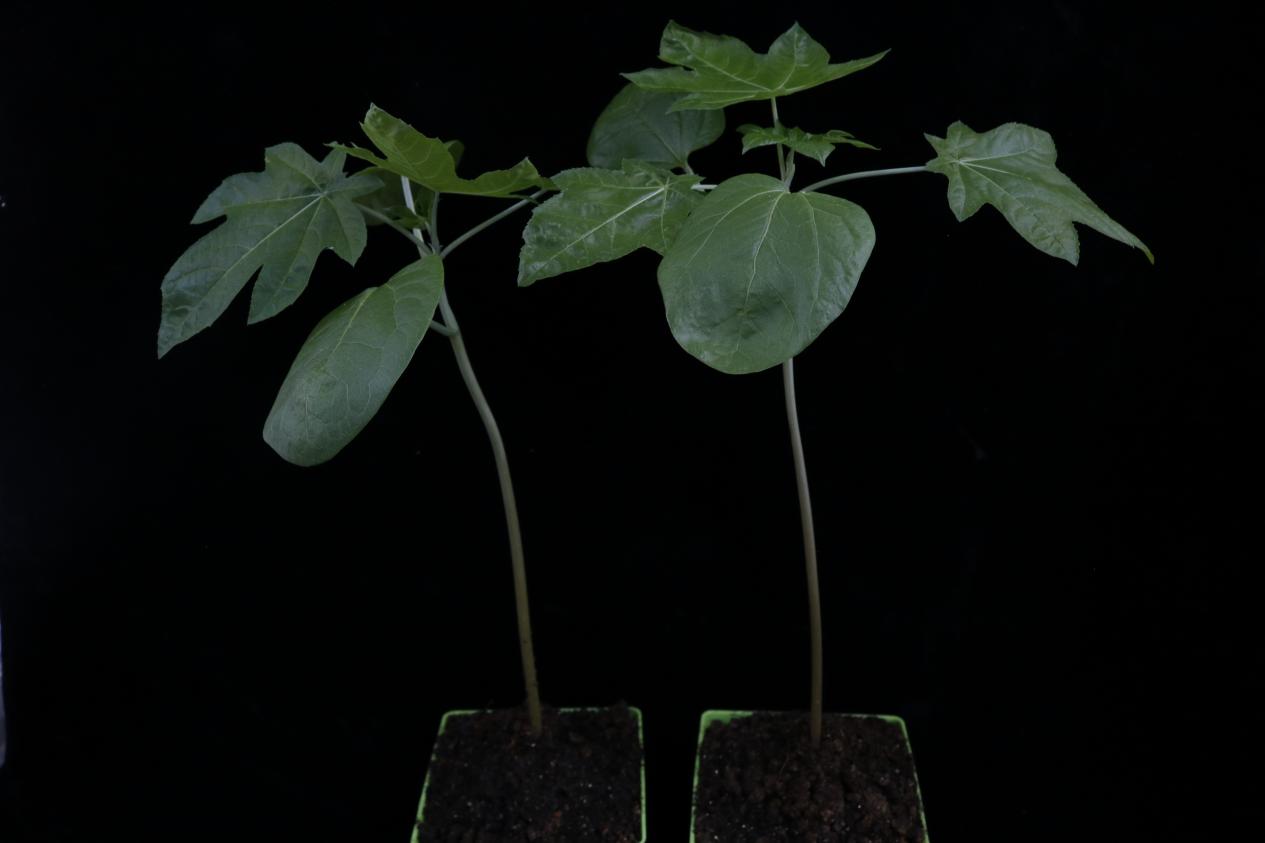


Figure S1. Castor experimental materials.


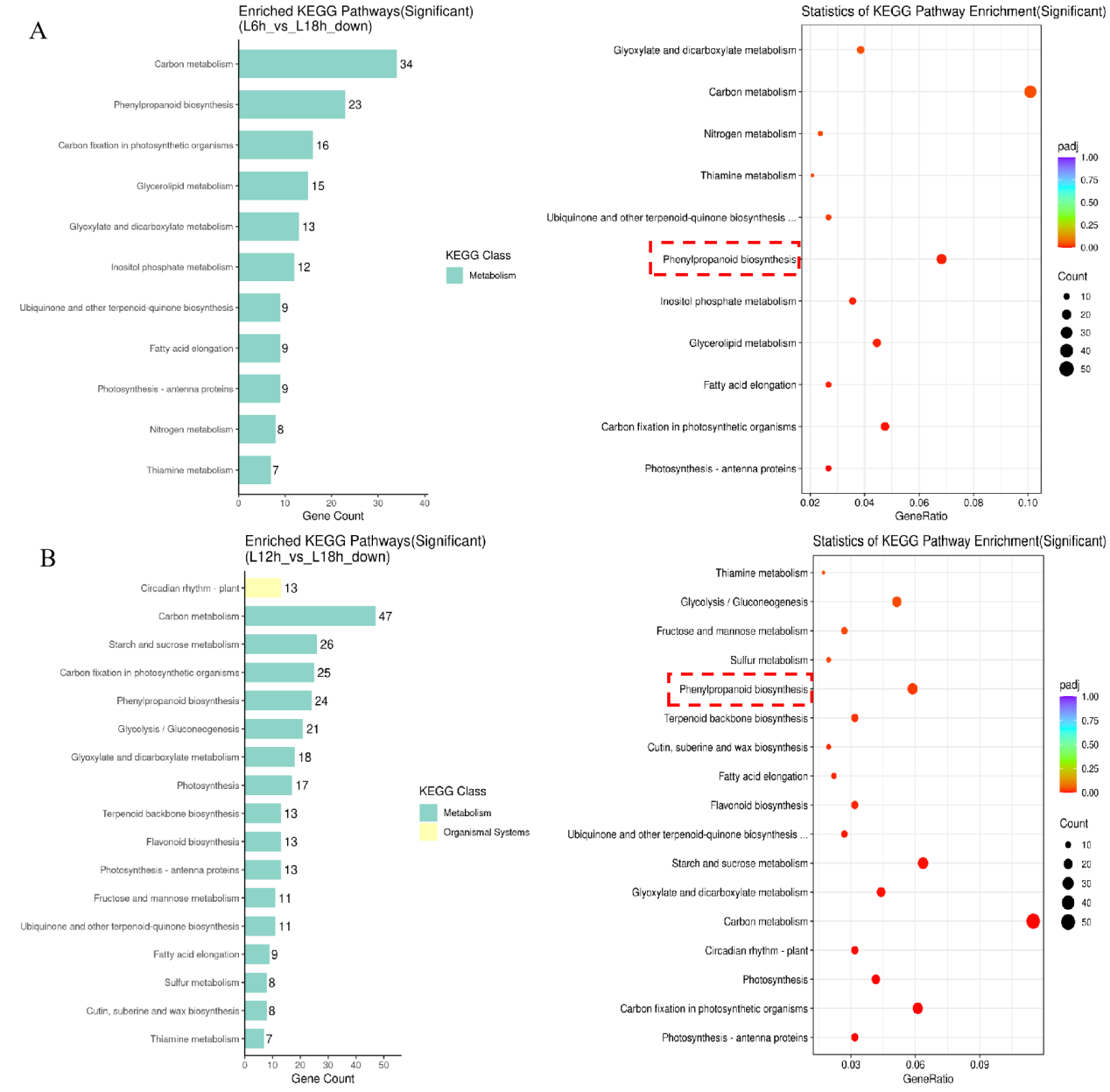


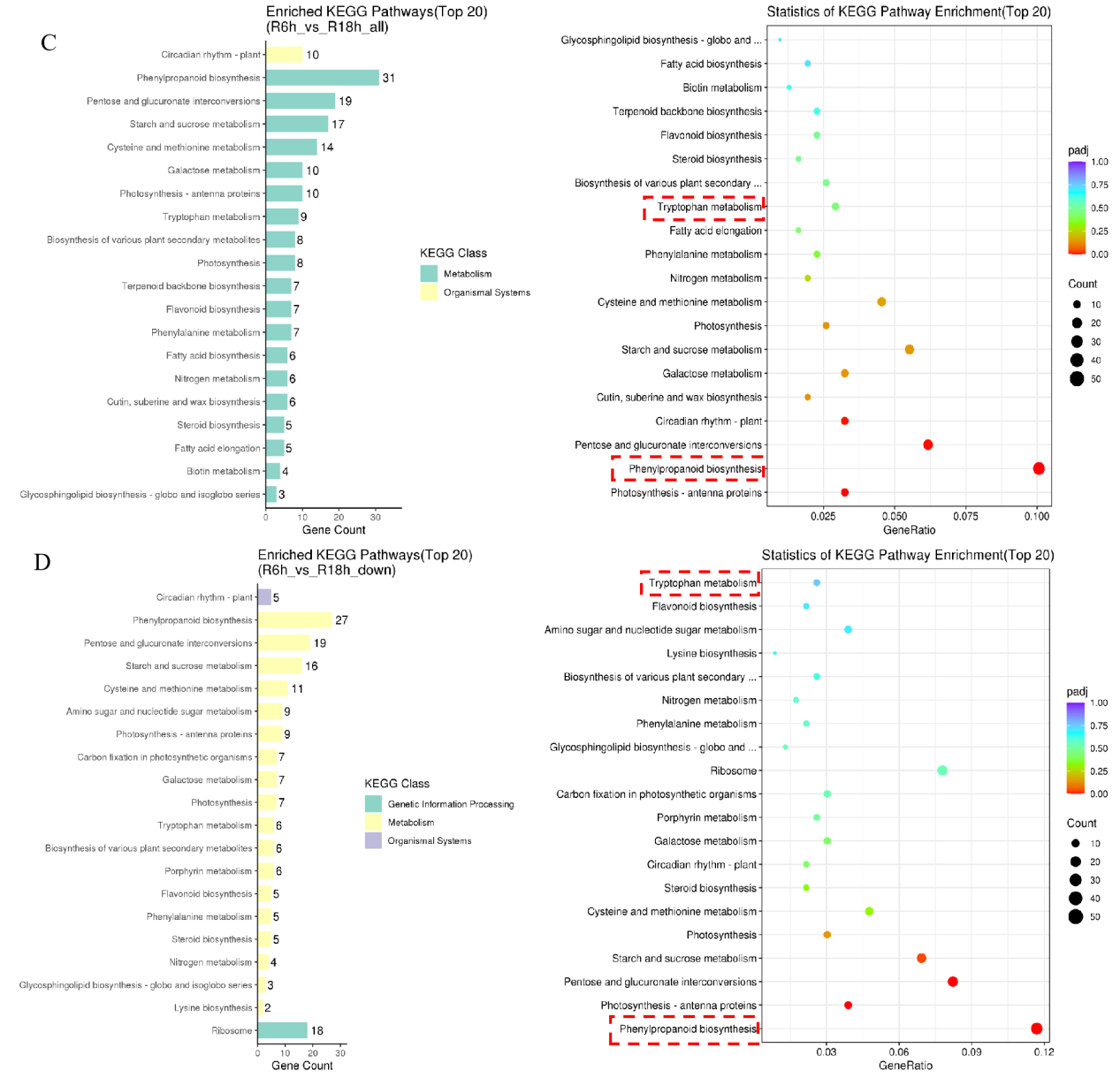


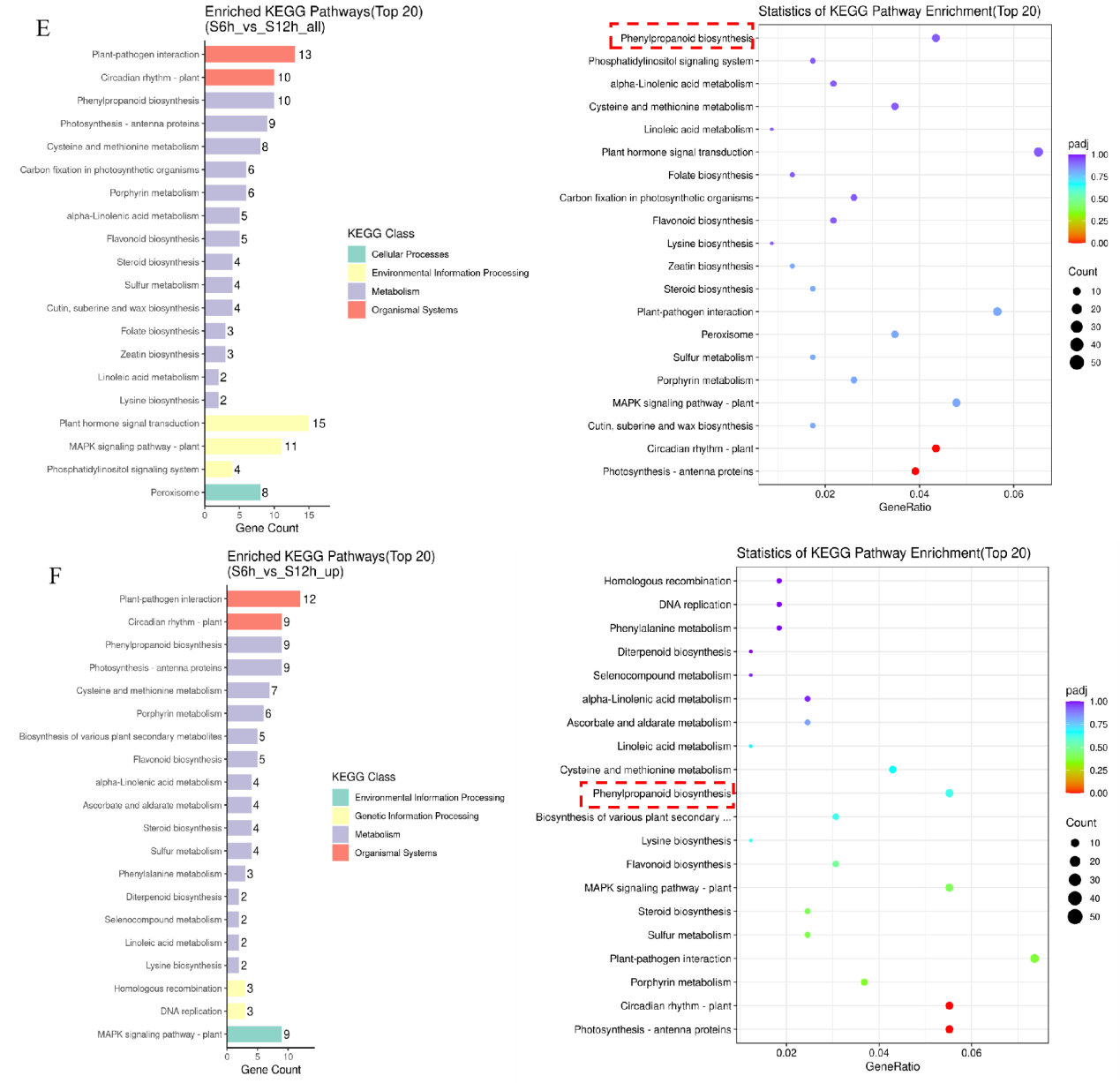


**Figure S2**. KEGG enrichment analysis of genes in castor at different tissues and time points. (A-B) Significant enrichment of phenylpropanoid biosynthesis was observed in the downregulated genes in leaf tissue at 6:00 vs 18:00, and 12:00 vs 18:00, respectively. (C-D) Significant enrichment of phenylpropanoid biosynthesis and enrichment of tryptophan metabolism were observed in the downregulated genes in root tissue at 6:00 vs 18:00. (E-F) Enrichment of phenylpropanoid biosynthesisgenes was observed in the upregulated genes in stem tissue at 6:00 vs 12:00.
